# Sensory innervation of the human palmar aponeurosis in healthy individuals and patients with palmar fibromatosis

**DOI:** 10.1111/joa.13609

**Published:** 2021-12-08

**Authors:** Irene García‐Martínez, Yolanda García‐Mesa, Jorge García‐Piqueras, Antonio Martínez‐Pubil, Juan L. Cobo, Jorge Feito, Olivia García‐Suárez, José A. Vega

**Affiliations:** ^1^ Servicio de Cirugía Plástica y Reparadora Fundación Hospital de Jove Gijón Spain; ^2^ Departamento de Morfología y Biología Celular Grupo SINPOS Universidad de Oviedo Oviedo Spain; ^3^ Instituto de Investigación Sanitaria del Principado de Asturias, and Hospital Universitario Central de Asturias Oviedo Spain; ^4^ Departamento de Cirugía y Especialidades Médico‐Quirúrgicas Universidad de Oviedo Oviedo Spain; ^5^ Instututo Asturiano de Odontología Oviedo Spain; ^6^ Servicio de Anatomía Patológica Hospital Clínico ‐ Complejo Hospitalario Universitario Salamanca Spain; ^7^ Facultad de Ciencias de la Salud Universidad Autónoma de Chile Santiago Chile

**Keywords:** acid‐sensing ion channel 2, Dupuytren's disease, mechanoproteins, palmar aponeurosis, palmar fibromatosis, PIEZO2, sensory corpuscles

## Abstract

The human palmar aponeurosis is involved in hand proprioception, and it contains different sensory corpuscle morphotypes that serve this role. In palmar fibromatosis (classically referred to as Dupuytren's disease), the palmar aponeurosis undergoes fibrous structural changes that, presumably, also affect the nervous system, causing altered perception. We analysed the various sensory nerve formation morphotypes in the palmar aponeuroses of healthy subjects and patients with palmar fibromatosis. To do this, we used immunohistochemistry for corpuscular constituents and the putative mechanoproteins PIEZO2 and acid‐sensing ion channel 2. Free nerve endings and Golgi‐Mazzoni, Ruffini, paciniform and Pacinian corpuscles were identified in both the healthy and the pathological conditions. The densities of the free nerve endings and Golgi‐Mazzoni corpuscles were slightly increased in the pathological tissues. Furthermore, the Pacinian corpuscles were enlarged and displayed an altered shape. Finally, there was also morphological and immunohistochemical evidence of occasional denervation of the Pacinian corpuscles, although no increase in their number was observed. Both PIEZO2 and acid‐sensing ion channel 2 were absent from the altered corpuscles. These results indicate that the human palmar aponeurosis is richly innervated, and the free nerve endings and sensory corpuscles within the palmar aponeurosis undergo quantitative and qualitative changes in patients with palmar fibromatosis, which may explain the sensory alterations occasionally reported for this pathology.

## Graphical abstract

The human palmar aponeurosis is densely innervated and contains a variety of sensory corpuscles as well as free nerve endings. Pacinian corpuscles express the mechanoproteins acid‐sensing ion channel 2 and PIEZO2. Palmar fibromatosis disease presents with increased densities of free nerve endings and Golgi‐Mazzoni corpuscles, but not of Pacinian corpuscles, although those that are present are enlarged and show morphological and immunohistochemical evidence of denervation.

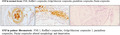

## 1 INTRODUCTION

Fasciae are important elements in muscle movement, motor coordination, mechanoception and postural regulation (Stecco, 2015; Stecco et al., 2011; Turrina et al., 2013); to fulfil these roles, they are richly innervated by nociceptive and mechanosensory nerve fibres. This applies to the upper arm fasciae (bicipital aponeurosis, antebrachial fascia and flexor retinaculum of the hand), which contain abundant free and encapsulated nerve endings (Stecco et al., 2007). Stecco et al. (2018) demonstrated that the palmar aponeurosis contains free nerve endings, Pacinian corpuscles and Golgi‐Mazzoni corpuscles, which are functionally related to different mechanoreception modalities (Cobo et al., 2020, 2021).

Retraction of digital and palmar aponeurosis caused by fibrous proliferation, known as Dupuytren's disease and Dupuytren's contracture, or better palmar fibromatosis (PF), is considered a fibroblastic/myofibroblastic tumour, as defined by the World Health Organization's Classification of Tumours of Soft Tissue and Bone (Goldblum & Fletcher, 2013). Thus, PF can be categorised within the superficial fibromatosis spectrum, together with Ledderhouse's disease in plantar aponeurosis and Peyronie's disease in penile aponeurosis (Montgomery et al., 2001).

In the context of PF, changes in the structure (Ehrmantant et al., 2004), number and size (Akyürek et al., 2000; Yenidunya et al., 2009) of Pacinian corpuscles have been reported. Stecco et al. (2018) recently observed that the density of free nerve endings in the palmar aponeuroses of patients with palmar fibromatosis is increased, but they did not provide information about changes to the other types of sensory corpuscles.

Mechanosensation is an essential component of somatosensitivity that conveys to the central nervous system mechanical inputs originating at the periphery of the body, including the skin, joints, ligaments, muscles, aponeurosis and fasciae. The sensory corpuscles and free nerve endings present in the palmar aponeurosis are related to mechanosensitivity, especially that involving the detection of vibration, shortening or elongation produced by mechanical deformations or pathology. Thus, they can be regarded as the peripheral organs of the mechanoreceptors. Interestingly, different ion channels, including PIEZO2 and acid‐sensing ion channel 2 (ASIC2), have been shown to be required for different mechanosensitivity modalities and have been detected in cutaneous mechanoreceptors and muscle proprioceptors (Cobo et al., 2020).

To the best of our knowledge, a systematic investigation of variations in the different sensory corpuscle antigens in patients with PF (Cobo et al., 2020, 2021; Vega et al., 2009) has not been conducted. Therefore, the aim of the present study was to better understand the sensory innervation of the human palmar aponeurosis and describe and characterise the somatosensory receptors located in the palmar aponeuroses of patients with PF compared to healthy subjects.

## 2 METHODS

### 2.1 Tissues

The tissues analysed in this study were obtained from selective fasciectomies of patients with PF (*n* = 13, 9 males and 4 females, age range 52–78 years), and they corresponded to both palmar (*n* = 5) and digital (*n* = 8) fasciae. The samples were obtained under informed consent from the patients from the Servicio de Cirugía Plástica y Reparadora of Hospital Universitario Central de Asturias and Fundación Hospital de Jove. Additionally, non‐pathological palmar aponeurosis tissues (*n* = 10, 3 males and 2 females, age range 65–80 years) corresponding to palmar (*n* = 5) and digital (*n* = 5) fascia were obtained from fresh cadavers during the removal of organs for transplantation (Hospital Universitario Central de Asturias). All the samples were obtained in compliance with Spanish Law and the guidelines of the Helsinki Declaration, and the study was approved by the Ethical Committee for Clinical Research of the Principality of Asturias, Spain (Reference 125/15).

The specimens were fixed in a solution of 10% formaldehyde in 1 M phosphate‐buffered saline (pH 7.6) for 24–48 h, after which they were dehydrated and embedded in paraffin. The pieces were then cut into 10‐µm‐thick sections and mounted on gelatine‐coated microscope slides. Finally, the selected sections were stained with haematoxylin–eosin and Masson trichrome to ascertain the structural details.

### 2.2 Single immunohistochemistry

Deparaffinised and rehydrated tissue sections were processed for immunohistochemical detection of proteins specific to the main corpuscular constituents and the mechanoproteins ASIC2 and PIEZO2 using the EnVision antibody complex detection kit (Dako) and following the manufacturer's instructions. Briefly, the endogenous peroxidase activity was inhibited (3% H_2_O_2_ for 15 min), and non‐specific binding was blocked (10% bovine serum albumin for 20 min). The sections were then incubated overnight at 4°C with the primary antibodies. The antibodies used were directed against the axon, Schwann‐related cells and endoneurial–perineurial cells (Cobo et al., 2021; Vega et al., 2009) (Table 1), and they included anti‐neurofilament protein (NFP, monoclonal, raised in mice, clone 2F11, prediluted antibody; Biocare Medical TM), anti‐S100β (polyclonal, raised in rabbits, used a 1:5000 dilution; DAKO), anti‐CD34 (monoclonal, raised in mice, clone QB‐END/10, pre‐diluted antibody; Master DiagnósticaTM), anti‐vimentin (monoclonal, raised in mice, clone 334, diluted 1:500; Boehringer‐Mannheim), anti‐ASIC2 (polyclonal, raised in rabbits, diluted 1:100; Lifespan Biosciences) and anti‐Piezo2 (polyclonal, raised in rabbits, diluted 1:200; Sigma‐Aldrich).

**TABLE 1:** Primary antibodies used in this study

Antigen	Origin	Dilution	Supplier
ASIC2	Rabbit	1:100	Lifespan Bioschences^a^
CD34 (clone QB‐END/10)	Mouse	Prediluted	Master Diagnostica^b^
NFP (clone 2F11)	Mouse	1:1000	Biocare Medical^c^
PIEZO2	Rabbit	1:200	Sigma‐Aldrich^d^
S100β protein	Rabbit	1:1000	Dako^e^
Vimentin (clone 3B4)	Mouse	1:500	Sigma‐Aldrich^d^

Abbreviations: ASIC2, acid‐sensing ion channel subunit 2; NFP, neurofilament protein; PIEZO2, piezo‐type mechanosensitive ion channel component 2.

^a^ Seattle, WA, USA.

^b^ Granada, Spain.

^c^ Concord, CA, USA.

^d^ Saint Louis, MS, USA.

^e^ Glostrup, Denmark.

For control purposes, representative sections were processed in the same way as described above using non‐immune rabbit or mouse sera instead of the primary antibodies or by omitting the primary antibodies during incubation. Under these conditions, positive immunostaining was not observed (data not shown). On the other hand, nerve trunks and fibres in the same sections served as internal positive controls for all the antigens investigated (Vega et al., 2009).

### 2.3 Double immunofluorescence staining

Since some studies devoted to the innervation of ligaments, fasciae and aponeuroses have shown confusing images of Ruffini corpuscles that resemble oblique sections of dermal nerves, we performed a double immunohistochemistry assay for the visualization of S100 proteins and myelin‐basic proteins (MBPs; clone SMI 99; Sternberger Monoclonals Inc.; diluted 1:500). Glial cells in sensory corpuscles never myelinate; therefore, the occurrence of MBP within a corpuscle‐like structure excludes it from being a sensory corpuscle. Non‐specific binding was reduced by incubation for 30 min in a 1% bovine serum albumin solution, followed by overnight incubation at 4°C in a humid chamber with a 1:1 mixture of rabbit anti‐S100 antibody and mouse anti‐MBP antibody (diluted 1:1000 and 1:200, respectively). After rinsing with TBS, the sections were incubated for 1 h with Alexa Fluor 488‐conjugated goat anti‐rabbit IgG (Serotec) that was diluted 1:1000 and then rinsed again and incubated for another hour with Cy_3‐conjugated donkey anti‐mouse antibody (Jackson ImmunoResearch) that was diluted 1:50. Both steps were performed at room temperature in a dark humid chamber. The sections were then washed, dehydrated and mounted using Entellan. Double staining was detected using a Leica DMR‐XA automatic fluorescence microscope coupled with Leica Confocal Software, version 2.5 (Leica Microsystems GmbH), and the captured images were processed using ImageJ version 1.43 g software (McMaster Biophotonics Facility, McMaster University, Ontario; http://www.macbiophotonics.ca/).

### 2.4 Quantitative analyses

The presence of free nerve endings and sensory corpuscles was investigated in five sections per histological sample that were separated by 100 µm. A total of 120 s processed for the detection of S100 protein were analysed, and they consisted of 25 healthy palmar aponeurosis sections, 25 healthy digital aponeurosis sections, 25 pathological palmar aponeurosis sections and 45 pathological digital aponeurosis sections. Each section was scanned with an SCN400F scanner (Leica Biosystems™) and annotated using SlidePath Gateway LAN software (Leica Biosystems™). A 4‐mm^2^ grid was then applied randomly onto 4 × 500 µm enlarged images in five non‐overlapping fields (20 mm^2^ per section and 100 mm^2^ per subject), and the free nerve endings and sensory corpuscles within the grid were counted by two independent observers. The results are expressed as absolute numbers for the densities of the sensory corpuscles and free nerve endings/cm^2^. Furthermore, the mean diameter of the Pacinian corpuscles was evaluated in the same sections, at the terminal zone of the corpuscle, and the values are expressed in millimetres.

The intensity of immunostaining developed with the axon, inner core and outer core–capsule complex of the Pacinian corpuscles was evaluated in arbitrary units of grey levels ranging from 1 (black) to 256 (white) using an image analysis system (MIP System, Servicio de Análisis de Imágenes, Universidad de Oviedo). The intensities were therefore divided into four groups (64 units of grey level each) referred to in the text and tables as strong (1–64, ++++), high (65–128, +++), intermediate (129–192, ++) and low (193 level of the background in control sections, +). Intensities of grey higher than those of the background of the corresponding control sections were considered as unreactive (– in table). We evaluated a total number of 89 Pacinian corpuscles (64 from normal palmar aponeurosis and 25 from palmar aponeurosis with fibromatosis), in which the axon, inner core and the complex outer core–capsule complex were delimited manually to measure grey intensity.

Significant differences among healthy and PF groups were assessed with the Kruskal‐Wallis *H* test, and *p* values of <0.05 were considered statistically significant.

## 3 RESULTS

The identification and classification of the sensory nerve formations in the palmar aponeuroses were based on Stecco et al. (2018), but we added another morphotype: the paciniform or simple lamellar corpuscles (Malinovský & Pac, 1982; Zelena, 1994).

### 3.1 Normal palmar aponeuroses

Various kinds of sensory nerve structures (either isolated free nerve fibres (endings) or differentiated sensory corpuscles) were identified in the non‐pathological palmar aponeuroses (both palmar and digital segments).

**FIGURE 1:**
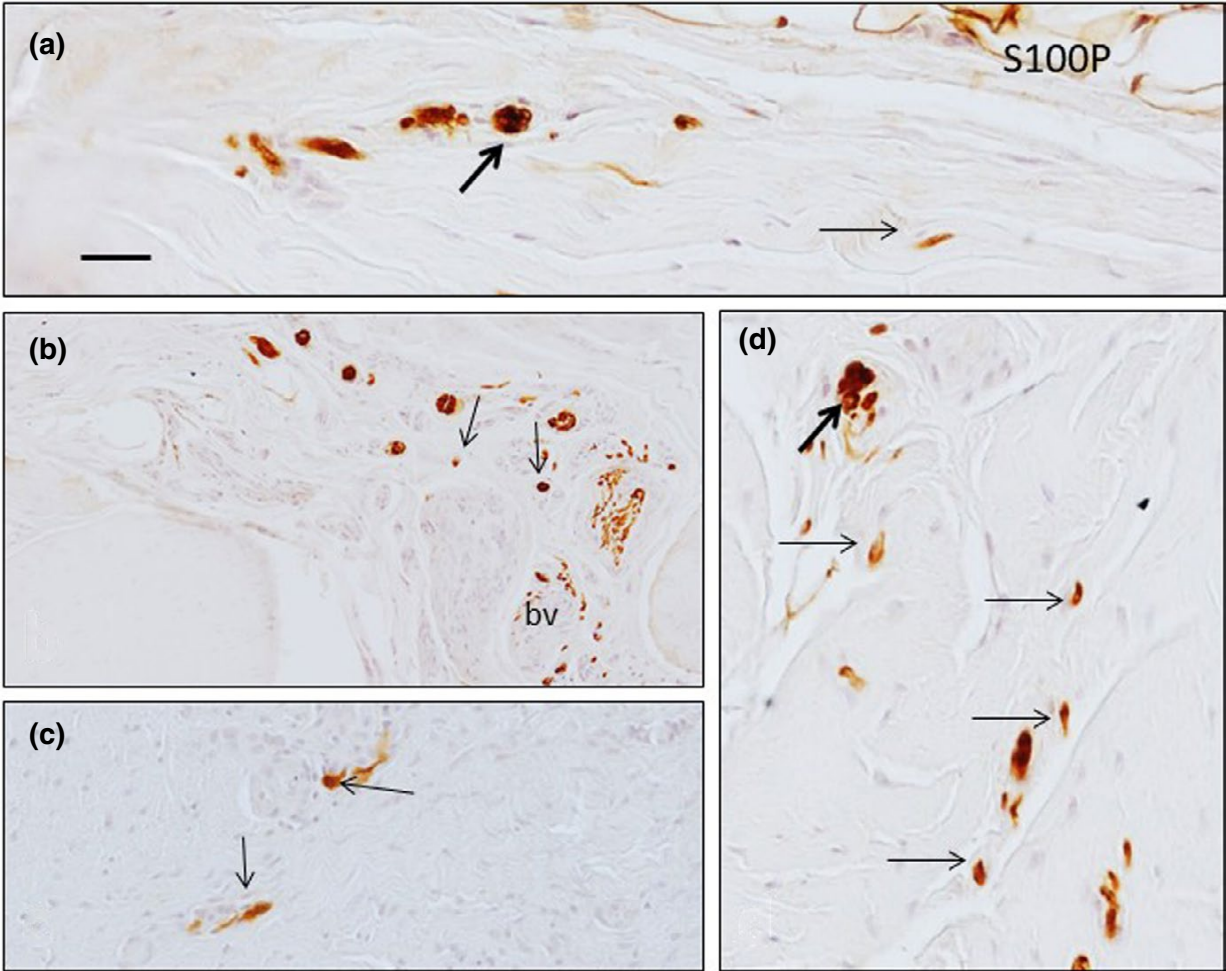
Sections of healthy human palmar aponeurosis processes for the detection of S100 protein (S100P) show the presence of small nerve bundles (thick arrows) and free nerve endings (thin arrows). Scale bar: 50 µm. bv: blood vessel

Small nerve bundles and free nerve fibres were identified amongst the connective tissue fascicles, and they displayed immunoreactivity to axonal markers and S100 protein (an axon is never nude; rather, it is covered by Schwann cell processes). No apparent regional differences were observed in the free nerve ending densities (Figure 1).

**FIGURE 2:**
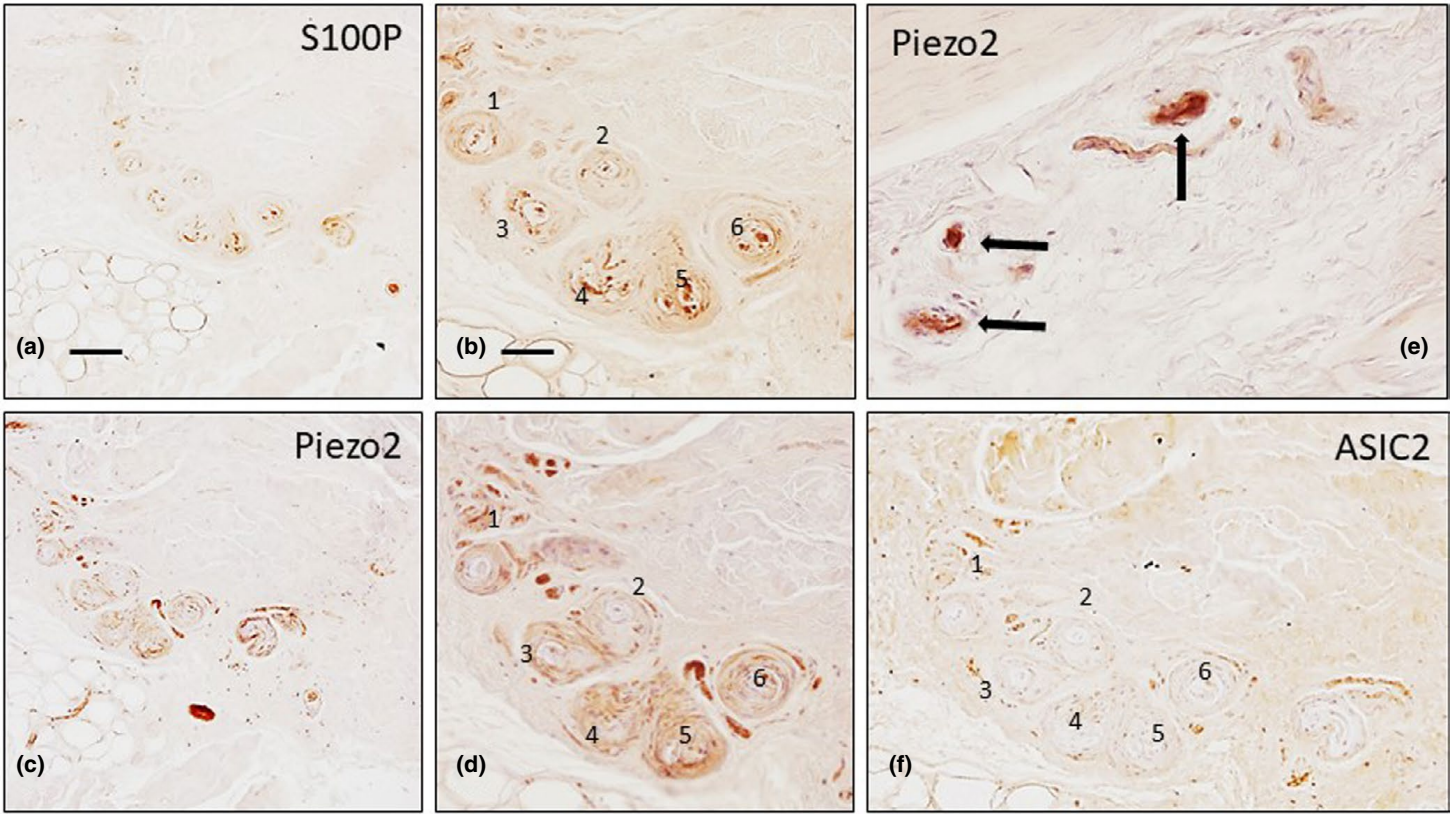
Serial sections of healthy human palmar aponeurosis processes for detection of S100 protein (S100P; a, b), Piezo2 (c–e) and ASIC2 (f). A group of paciniform sensory corpuscles (numbered 1–6) displays S100P immunoreactivity in the inner core cells and PIEZO2 immunoreactivity in the inner core and capsule, but they lack ASIC2 immunoreactivity. Scale bar: 100 µm (a, c), 50 µm (b, d, e, f)

**FIGURE 3:**
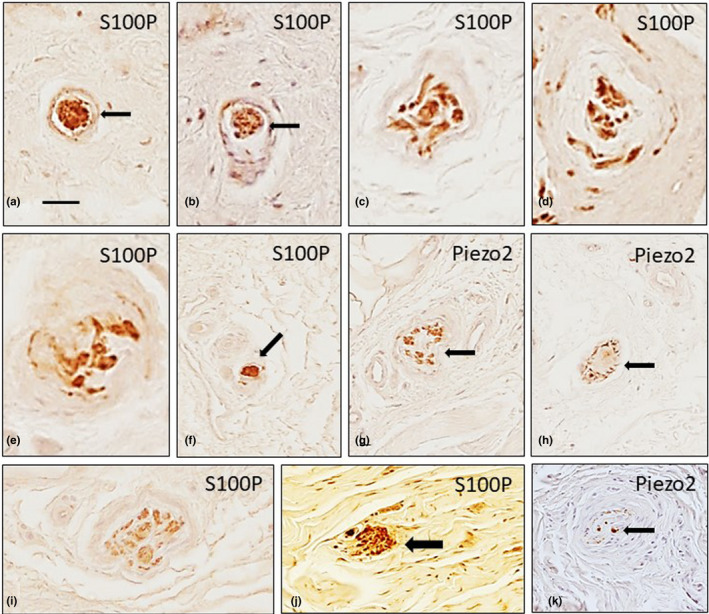
Different morphotypes of sensory corpuscles from healthy human palmar aponeuroses show immunoreactivity for S100 protein (S100P; a–f, i, j) and Piezo2 (g, h, k). Paciniform corpuscles (a, b, k; arrows) and Golgi‐Mazzoni corpuscles (c–j; arrows) were regularly found. Scale bar: 50 µm (a, b, f–k), 30 µm (c–e)

Capsulated and non‐capsulated sensory nerve formations were found throughout each palmar aponeurosis sample, with wide variations, although these variations did not appear to correlate with age, morphology, size or the arrangement of the neural elements (i.e., the axon‐ and Schwann‐related cells). Nevertheless, these formations can be regarded as some of the sensory corpuscles described previously in the fasciae and ligaments (Zelená, 1994). Paciniform or simple lamellar corpuscles were observed regularly (Figure 2). The inner core displayed strong immunoreactivity for S100 protein (Figure 2a–d), whereas the axon was immunoreactive for axonal markers and PIEZO2 (Figure 3a,b). These corpuscles lacked ASIC2 immunoreactivity (Figure 2f).

**FIGURE 4:**
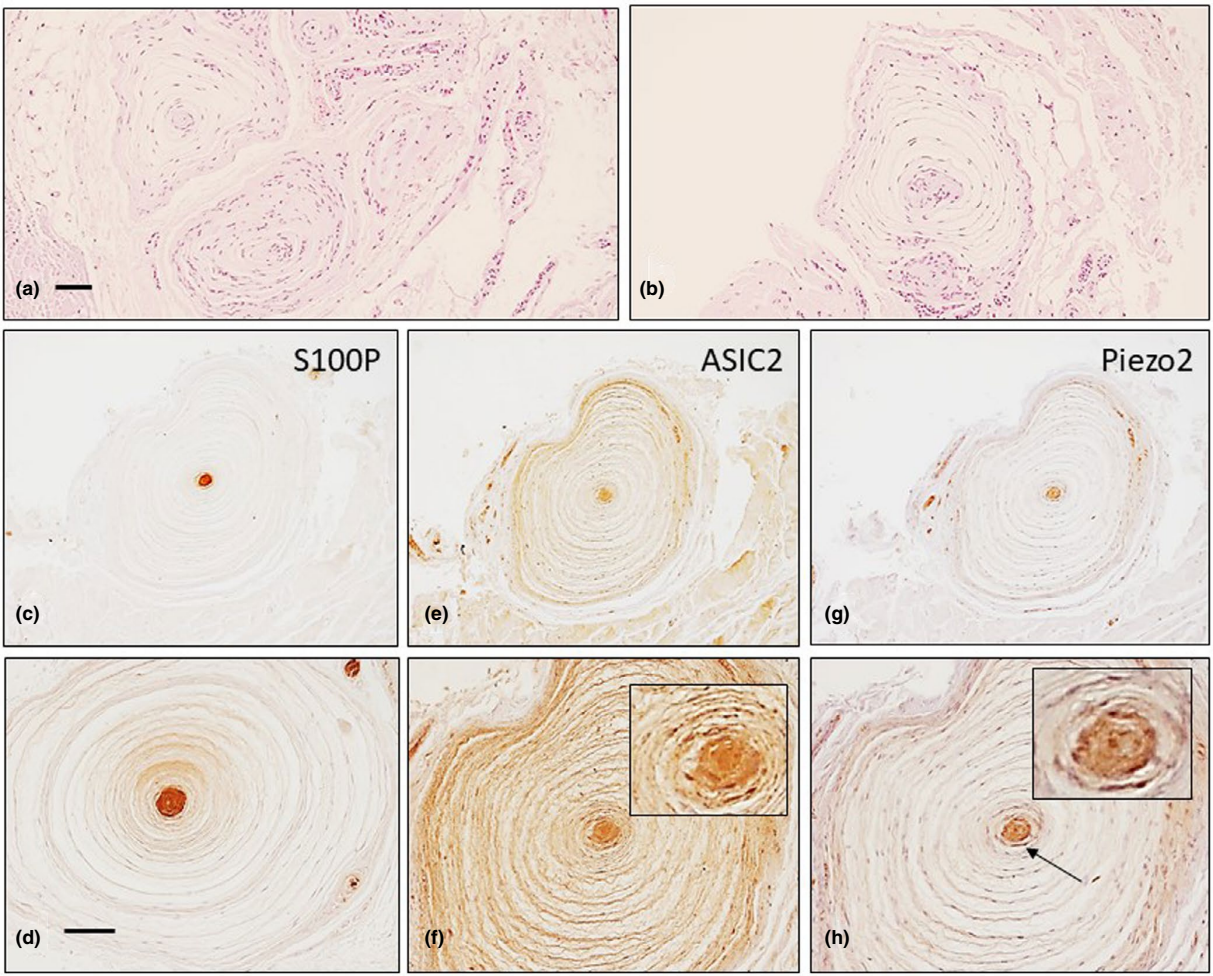
Pacinian corpuscles (isolated or in clusters) were found in healthy human palmar aponeuroses (a, b) Serial sections of Pacinian corpuscles show localisation of S100 protein (S100P, c, d), ASIC2 (e, f) and Piezo2 (g, h). The arrow in h indicates the axon. Scale bar: 1 mm (a–g), 300 µm (d–h)

Stretched and irregularly shaped Golgi‐Mazzoni corpuscles were also observed (Figure 3). The neural apparatus was variably branched and embedded in an acellular substance (Figure 3c–e, i and j) or at the periphery of the central bulb (Figure 3g,h). No typical Ruffini corpuscles were observed. Some densely packed lamellar bodies that were covering a group of axons were also identified and interpreted as simple lamellar corpuscles (Figure 3k). Abundant typical Pacinian corpuscles, either isolated or in groups of two to four corpuscles, were observed (Figure 4a,b). The central axon displayed immunoreactivity for the axonal markers, the lamellae forming the inner core showed strong S100 protein expression (Figure 4c,d), the intermediate layer was CD34 positive, and the inner and outer cores and the capsule were vimentin positive (Cobo et al., 2021). ASIC2 was detected in the inner core (Figure 4f), whereas PIEZO2 labelled both the axon and the inner core (Figure 4h).

### 3.2 Palmar aponeuroses in patients with PF

**FIGURE 5:**
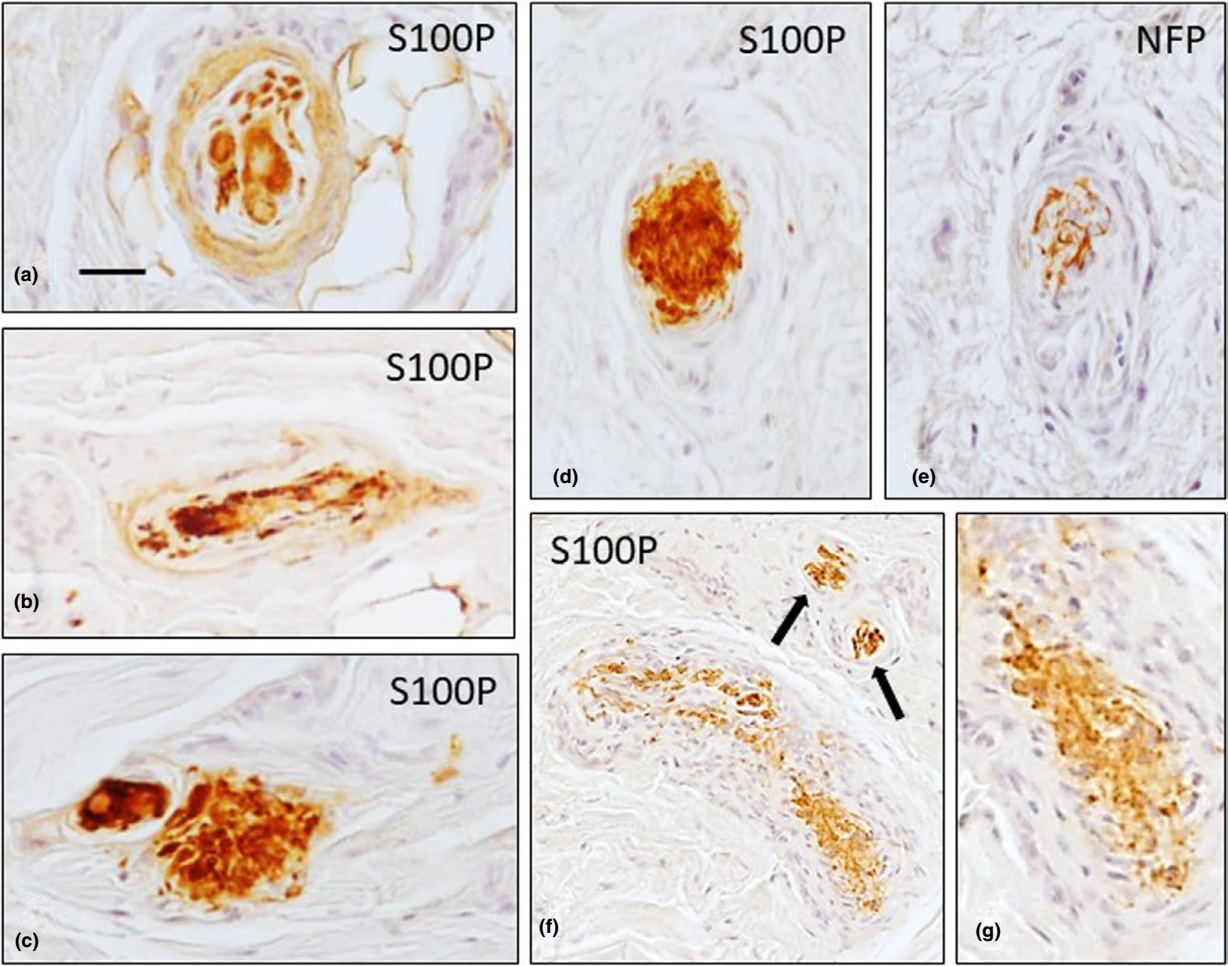
Different sensory corpuscle morphotypes in the palmar aponeuroses of palmar fibromatosis patients show immunoreactivity for S100 protein (S100P). The morphotypes include paciniform (a, b), Golgi‐Mazzoni (c–e; arrows) and Ruffini (f, g) corpuscles. The distribution of S100P and NFP in a Golgi‐Mazzoni corpuscle can be observed in the serial sections (d, e). Scale bar: 50 µm

Numerous free nerve endings were observed coursing amongst the collagen fibres. Additionally, the sensory corpuscle morphotypes encountered in the PF samples were similar to those reported in non‐pathological aponeuroses. These included paciniform corpuscles with single or multiple inner cores (Figure 5a,b) and Golgi‐Mazzoni and Ruffini‐like corpuscles with differently developed capsules (Figure 5c–e). Furthermore, typical Ruffini's corpuscles were identified in one sample (Figure 5f,g). None of these morphotypes of corpuscles showed morphological changes or immunohistochemical variations compared to those found in the normal aponeuroses. Nevertheless, a significant increase (*p* < 0.05) in free nerve endings was encountered.

**FIGURE 6:**
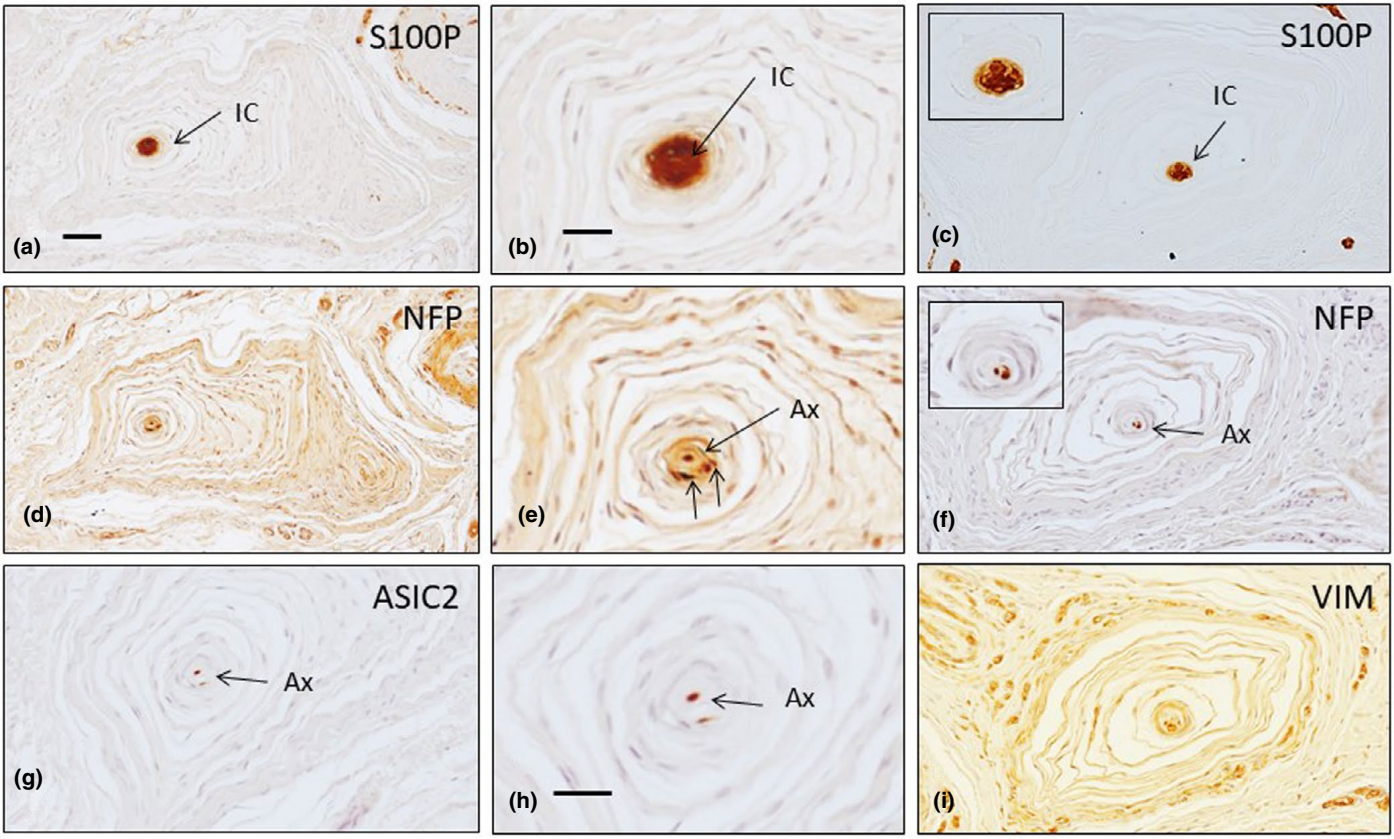
Serial sections of Pacinian corpuscles in palmar fibromatosis patients show immunoreactivity for S100 protein (S100P; a–c), neurofilament proteins (NFP; d–f; arrows indicate axons), ASIC2 (g, h; arrows indicate axons) and vimentin (VIM, i). Ax: axon, IC: inner core. Scale bar: 1 mm (a, c, e, g–i), 300 µm (b, d, f)

**FIGURE 7:**
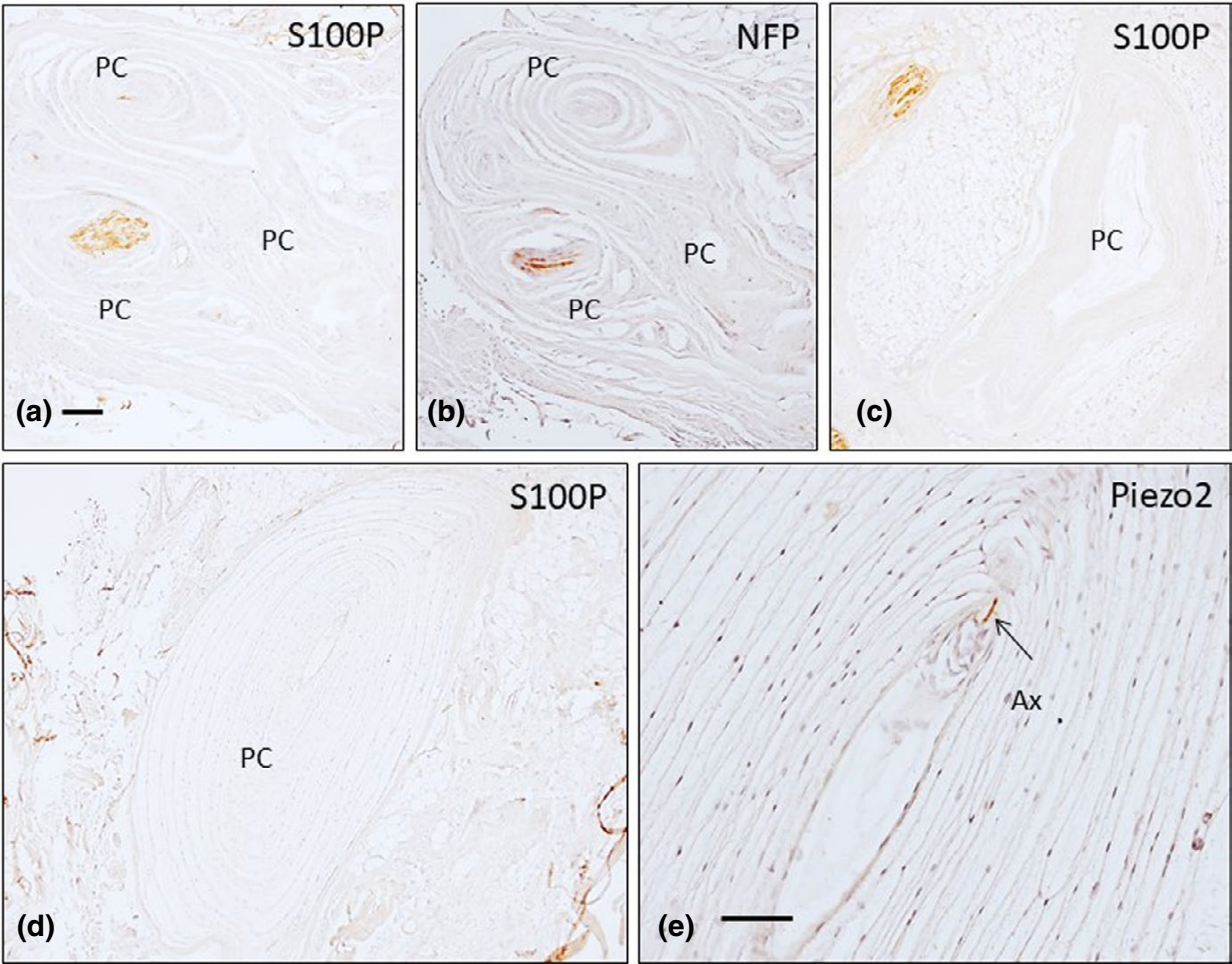
Serial sections of Pacinian corpuscles from palmar fibromatosis patients show immunoreactivity for S100 protein (S100P; a, c, d), neurofilament proteins (NFP; b; arrows indicate axons) and PIEZO2 (e; arrow indicates the axon). Ax: axon, PC: Pacinian corpuscle. Scale bar: 1 mm (a–d), 300 µm (e)

**FIGURE 8:**
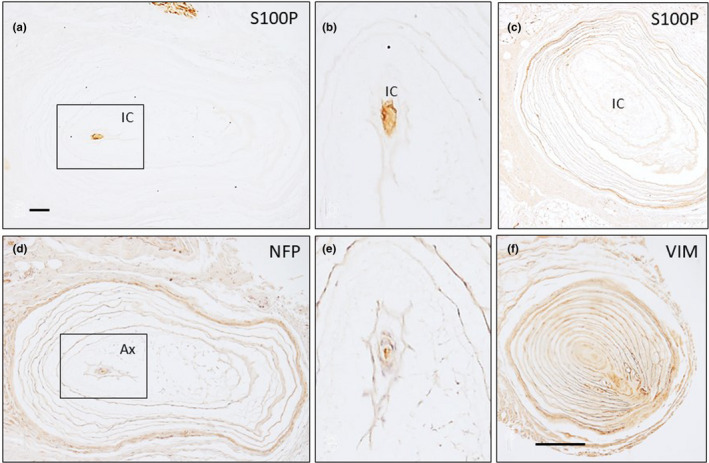
Sections of Pacinian corpuscles from palmar fibromatosis patients show immunoreactivity for S100 protein (S100P; a–c), neurofilament proteins (NFP; d, e) and vimentin (VIM, f). Ax: axon, IC: inner core. Scale bar: 1 mm (a, c, d, f), 300 µm (b, e)

Approximately 35% of the PF Pacinian corpuscles retained the typical structure and immunohistochemical profile. The remaining 65% showed different changes in the morphology and the investigated antigens: 33% showed normal structure and size but retained immunoreactivity at residual levels; 14% showed normal structure and size but antigens identifying the axon, capsule or inner core were absent; 7% were hypertrophic and retained immunoreactivity at residual levels and 11% were hypertrophic and unreactive for the antigens investigated (Figures 6 and 7). The mean diameter was 2.32 ± 0.31 (range 3.6–0.64 mm) in PF versus 1.28 ± 0.31 mm (range 0.6–2.1 mm) in controls. No evidence of hyperplasia (groups of corpuscles) was observed. Furthermore, only a small percentage of Pacinian corpuscles (approximately 5%) displayed immunoreactivity for ASIC2 and PIEZO2 proteins (Figure 8).

The results of the quantitative study are summarised in Table 1, whereas those of the qualitative study are summarised in Table 2. No significant differences in the densities of the sensory nerve formations were found between the normal subjects and the PF patients, with the exception of free nerve endings which were increased (Table 3).

**TABLE 2:** Densities of the free nerve endings and sensory corpuscles in the normal fascia palmaris patients and patients with palmar fibromatosis

	Normal palmar aponeurosis (per cm^2^)	Palmar aponeurosis in fibromatosis (per cm^2^)
Free nerve endings	9.2	12.1*
Ruffini corpuscles	Not found	1**
Golgi‐Mazzoni corpuscles	5.3	6.3
Paciniform and lamellar simple corpuscles	4.4	4.1
Pacinian corpuscles	3.2	3.6

* *p* < 0.05.

** One unique typical Ruffini corpuscle was identified in all the sections analysed.

**TABLE 3:** Changes in the corpuscular antigens investigated in the Pacinian corpuscles from the palmar aponeuroses of the normal subjects and patients with palmar fibromatosis

	Cell specificity	Normal fascia palmaris	Fascia palmaris in fibromatosis
Neurofilament proteins	Axon	++++ 100%	++++ 35% ++ 16% + 24% −25%
S100 protein	Inner core	++++ 100%	++++ 35% ++ 10% + 30% −25%
CD−34	Intermediate layer	+++ 100%	+++ 35% ++ 18% + 22% −25%
Vimentin	Outer core‐capsule	++++ 100%	++++ 35% ++ 25% + 15% −25%
ASIC2	Inner core	+++ 96%	++/+ 5% −95%
PIEZO2	Axon/Inner core	+++ 100% / ++ 92%	++/+ 5% −96%

Note

Strong (++++), high (+++), intermediate (++), low (+) or negative (−) immunoreactivity.

**FIGURE 9:**
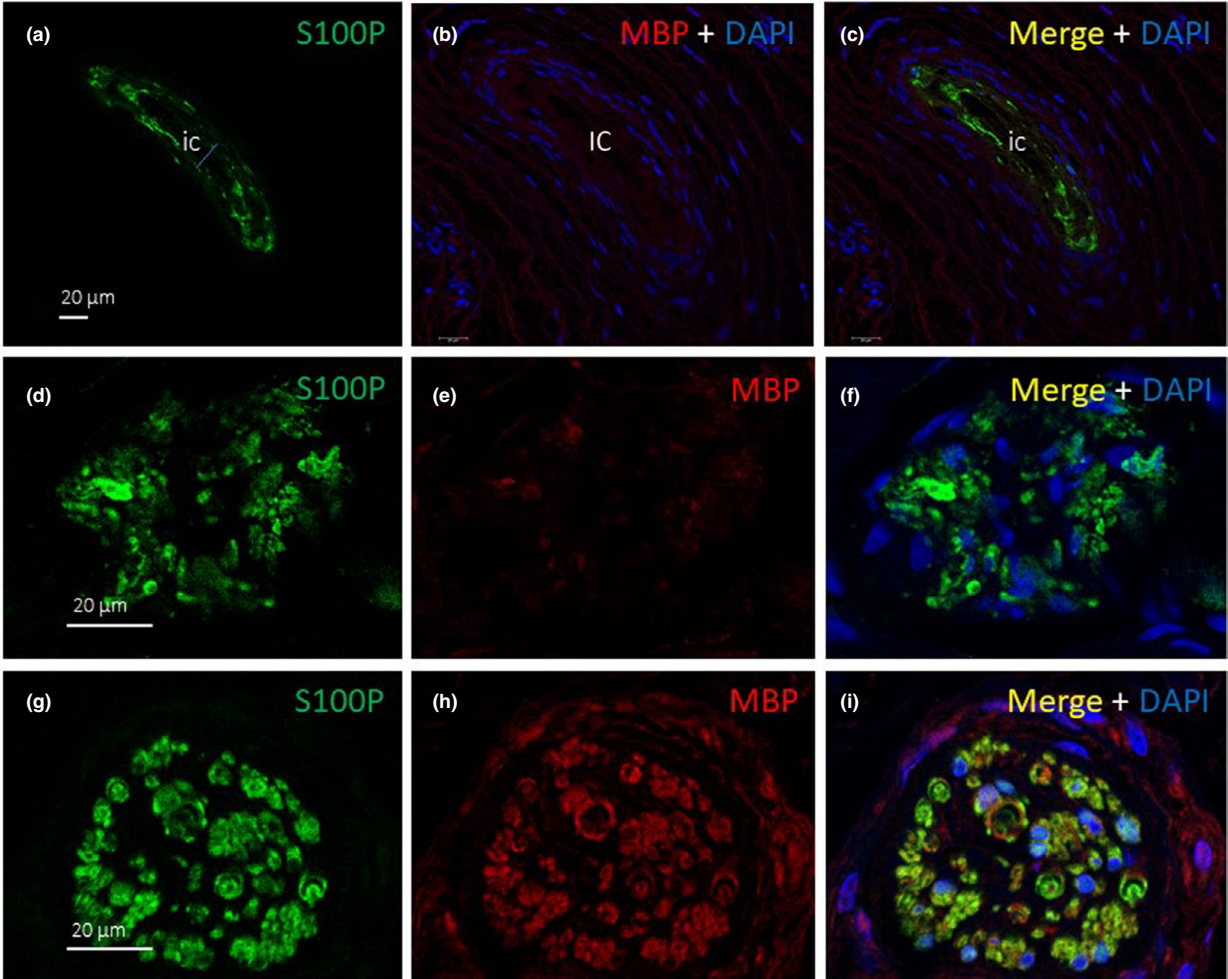
Double immunofluorescence of S100 protein (S100P, green fluorescence) and myelin basic protein (MBP, red fluorescence) in Pacinian (a–c) and Ruffini (d–f) corpuscles and a large nerve (g–i). Myelin basic protein was always absent in the morphologically differentiated sensory corpuscles, but it was co‐localised with S100 protein in the Schwann cells of nerve bundles. Sections were counterstained with DAPI to ascertain the structural details. ic: inner core; Objective 63×/1.40 oil; pinhole 1.37; XY resolution 139.4 nm and Z resolution 235.8 nm

MBP immunofluorescence was not detected in the Pacinian and Ruffini corpuscles; however, MPB and S100P were co‐localised in the large nerve fibres (Figure 9).

## 4 DISCUSSION

PF is a common superficial fibromatosis that affects digital and palmar aponeuroses. It is characterised by painless nodules located along longitudinal lines of tension; the nodules form cords that produce contracture deformities within fascial bands and tissues of the hand (Mella et al., 2018). Although most patients experience discomfort, pain is only an occasional side effect in the initial stages (von Campe et al., 2012). However, pain is probably the extreme pole of a disease with an important peripheral nervous system involvement, as reduced sensitivity, likely due to peripheral and entrapment neuropathy, has been found in PF patients (Gerosa et al., 2018).

In the present study, we observed different types of sensory corpuscles in normal and pathological palmar aponeuroses. Our results are consistent with those of Stecco et al. (2018) regarding the types of sensory corpuscles present in normal palmar aponeuroses as well as the density of these formations. The small discrepancies are likely related to the number of specimens studied.

Additionally, we observed an increase in the number of free nerve endings in the palmar aponeuroses of PF subjects compared to those of normal patients, which is also in agreement with Schubert et al. (2006) and Stecco et al. (2018). Slight increases were also noted in the Golgi‐Mazzoni corpuscles. Nevertheless, because of the variation between individuals and the limited number of specimens investigated, these observations should be interpreted with caution.

Pacinian corpuscles, including those of the palmar aponeurosis, are well understood, both morphologically and antigenically (Cobo et al., 2021); Pacinian corpuscles in PF patients are essentially similar to those of normal patients, although some differences are readily apparent. In contrast to other studies (Akyürek et al., 2000; von Campe et al., 2012), we did not find evidence of Pacinian corpuscle hyperplasia in the palmar aponeuroses of patients with PF. Instead, we observed enlarged Pacinian corpuscles, which is consistent with the reports from Ehrmantant et al. (2004) and Yenidunya et al. (2009). Ehrmantant et al. (2004) observed that the Pacinian corpuscles of PF patients had significantly more layers. They did not, however, describe whether the additional layers involved the outer core capsule, intermediate layer, inner core or all these structures. Furthermore, they related these corpuscular changes to an over‐synthesis of nerve growth factor, as human cutaneous and articular Pacinian corpuscles express the high‐affinity signalling receptor for this neurotrophin (Del Valle et al., 1998; Vega et al., 1994).

In contrast to previous studies (Ehrmantant et al., 2004; Yenidunya et al., 2009), we observed that approximately 65% of the PF Pacinian corpuscles showed signs of partial or total denervation (Zelená, 1994). Our findings are in line with those of Mikusev (1989), who described the 'deformation' of Pacinian corpuscles and degeneration of nerve fibres in the first stages of PF, followed by the loss of Pacinian corpuscles in later stages. In fact, PF corpuscles lack immunoreactivity to neuronal markers, indicating rearrangement, reduction, or even the absence of inner core cells, all of which are typical features of corpuscular denervation (Albuerne et al., 1998; Márquez et al., 1997). Furthermore, Pacinian corpuscles from PF patients lack immunoreactivity for PIEZO2 and ASIC2, as well (Anderson et al., 2017; Cabo et al., 2015; Delmas & Coste, 2013). The activation of mechanically gated ion channels marks the onset of low‐and high‐threshold mechanical stimuli detection. Multiple candidates have been proposed as mechanotransducers in human cutaneous sensory corpuscles; however, only PIEZO2 is an essential component of distinct stretch‐activated ion channels involved in mechanotransduction (Cobo et al., 2020). In the current study, PIEZO2 immunoreactivity was detected in all the different types of sensory corpuscles and the free nerve endings, which lends support to the mechanosensory role of the corpuscles present in the palmar aponeurosis. Both ASIC2 and PIEZO2 were nearly completely absent from the sensory nerve formations in the patients with PF, suggesting that mechanotransduction is affected in these individuals. Structural and immunohistochemical changes in Pacinian corpuscles may be related to occupational exposure to hand‐transmitted vibrations and/or chronic trauma (Palmer et al., 2014). This remains to be confirmed in future studies, however.

The sensory nerve formations found in our study fall into two categories: free nerve endings that are primarily regarded as nociceptors (Wilke et al., 2017) and different sensory corpuscle morphotypes that likely work as mechanoreceptors. What mechanosensation modality they detect remains to be elucidated. The mechanoreceptors that are situated in the joint, including the joint capsule, ligaments, muscles, tendons and skin, all contribute to proprioceptive function (Blecher et al., 2018; Macefield, 2005); however, the muscle spindle provides the greatest contribution (Macefield & Knellwolf, 2018).

Although the results obtained are of interest because they allow us to know changes in sensory innervation in the course of FP, especially in the structure of the sensory corpuscles, the study has some limitations. Firstly, we consider that it is necessary to analyse whether the observed changes are equal in the palm of the hand and the fingers and also to establish whether changes in the peripheral nervous system progress in parallel with the formation of fibromatosis and finally, although we attempted to process all samples identically, there may be variations in the intensity of final immunoreaction due to technical aspects, that is differences in the penetration of the fixative.

In summary, PF courses with scarce changes to the innervation of palmar and digital aponeuroses. Nevertheless, Pacinian corpuscles in PF patients are frequently denervated (up to 65%), and no evidence of functional mechanotransduction is present. Our findings are in accordance with the natural history of the disease proposed by Mikusev (1989), in that partial denervation occurs at disease onset, and Pacinian corpuscles undergo hypertrophy before they completely atrophy and disappear during the later phases of the disease. Taken together, these variations might be correlated with the altered sensitivity reported in these patients (von Campe et al., 2012).

## CONFLICT OF INTEREST

There are no conflicts of interest to declare.

## AUTHOR CONTRIBUTIONS

IG‐M, YG‐M, JG‐P and AM‐P performed the experiments. IG‐M, JLC, JF and OG‐S collected the material in compliance with ethical guidelines and performed part of the experiments. JG‐P and JAV quantified the data. JF, OG‐S and JAV designed the study, analysed the data and wrote the manuscript.

## Data Availability

The data that support the findings of this study are available from the corresponding author upon reasonable request.
